# The Effect of Low-Level Laser Therapy to Reduce Pain Caused by Orthodontic Separators: A Randomized, Double-Blind Placebo-Controlled, Split-Mouth Study

**DOI:** 10.3390/dj13050181

**Published:** 2025-04-22

**Authors:** Alireza Khandan Dezfully, Márió Gajdács, Aliz Eperke Pató, Krisztina Kárpáti, Melinda Madléna

**Affiliations:** 1 Department of Orthodontics and Pediatric Dentistry, Faculty of Dentistry, University of Szeged, Tisza Lajos krt. 64-66., 6720 Szeged, Hungarykarpati.krisztina@stoma.szote.u-szeged.hu (K.K.); 2 Department of Oral Biology and Experimental Dental Research, Faculty of Dentistry, University of Szeged, Tisza Lajos krt. 64-66., 6720 Szeged, Hungary; gajdacs.mario@stoma.szote.u-szeged.hu

**Keywords:** orthodontics, orthodontic pain, low-level laser therapy, LLLT, laser, oral health, mandible

## Abstract

**Background:** During the initiation of routine orthodontic treatment with fixed appliances, placing elastic separators (ESs) may lead to the patient experiencing considerable pain. Earlier studies evaluating the effects of low-level laser therapy (LLLT) on reducing pain caused by orthodontic ESs have reported contradictory results. The aim of our study was to evaluate the effect of a single application of LLLT on the experience of pain following ES placement. **Methods:** A double-blind, placebo-controlled randomized controlled trial was performed—with implementation of the split-mouth technique—where *n* = 33 volunteers (12 male and 21 female; aged between 19 and 36 years) were enrolled. ESs were placed at the mesial and distal surfaces of the first permanent molars in the upper or lower jaws. Jaws were divided into two quadrants; the treatment group received LLLT (using a GaAlAs diode laser, at a 980 nm wavelength, with 100 mW producing 6 J of energy; continuous wave), while the other quadrant served as the placebo side receiving a similar treatment, but without laser irradiation, immediately after separation. A questionnaire with a visual analogue scale (VAS; 0–100) was used for the assessment of pain (spontaneous pain and pain on mastication) scored for each first permanent molar directly after separation and after 6, 24, 48 and 72 h of both laser and placebo treatment application. **Results:** Mean VAS values were lower, but not significantly different, between the treatment vs. placebo groups for spontaneous pain at either measurement point (*p* > 0.05). Mean VAS values were significantly lower in the treatment groups for pain on mastication at the 6 (9.29 ± 12.65 vs. 19.07 ± 20.99; *p* = 0.025), 24 (21.96 ± 21.11 vs. 37.19 ± 26.35; *p* = 0.012) and 48 h (28.01 ± 21.40 vs. 42.60 ± 26.29; *p* = 0.016) measurement points. The analgetic effect of LLLT was most effective after 6 h, both in the resting position (−49% decrease) and during mastication (−51% decrease). **Conclusions:** A single application of LLLT with 6 J of energy may have beneficial effects on reducing the pain caused by orthodontic ESs used at the initiation of treatment with fixed appliances, especially during mastication, after 6–48 h following the separation procedure. **Trial registration:** clinicaltrials.gov ID NCT06816537 (date of registration: 7 February 2025, retrospectively registered).

## 1. Introduction

After finishing the development of the jaws, the effective treatment of orthodontic anomalies needs fixed appliances. During the initiation of routine orthodontic treatment with fixed appliances, placing elastic separators (ESs) is a part of the process to achieve an adequate space for orthodontic bands, which may lead to patients experiencing considerable pain [1]. Previous studies have shown that 90–95% of patients reported various degrees of pain, and 8–30% out of them were not able to continue orthodontic treatment [1,2,3,4]. It was shown that this experience of pain is a key barrier to the completion of orthodontic treatment processes [5]. Therefore, different approaches have been considered to reduce the pain due to orthodontic treatment, such as cognitive behavioral therapy (CBT) [6], chewing gum or soft/hard viscoelastic wafers [7] and pharmacological management using medications (i.e., ibuprofen) or topical anesthetic gels [1,8]. Nonsteroidal anti-inflammatory drugs (NSAIDs) can reduce orthodontic pain; however, they may negatively affect the tooth movement process by inhibiting bone resorption, and they may also have various other adverse effects on some patients (e.g., xerostomia) [9].

In recent times, dental laser therapy has received substantial attention in orthodontics, especially from the point of view of tooth movement, root resorption, the stability of miniscrews or affecting the sensation of pain [10]. According to their intensity and indicated uses in dentistry, lasers may be classified as high-intensity/level laser therapy (HILT/HLLT) and low-level laser therapy (LLLT) [11]. HILT lasers usually operate above the 500 mW range and between the wavelengths of 810 and 1064 nm, being most commonly used in dentistry for atraumatic surgical interventions (e.g., frenectomy, fibretomy and gingivectomy), due to the advantageous clinical outcomes associated with these procedures [12,13]. On the other hand, the main applications of LLLT include the stimulation of tissue-healing and regenerative processes, using laser devices operating below 500 mW [11,13]. Most recently, LLLT—during which the energy output of the laser is sufficiently low to prevent a temperature rise above 36.5 °C (normal body temperature) in the target tissue—may be used as a convenient analgesic therapy for orthodontic patients [14,15,16,17,18,19,20,21,22]. LLLT may be a useful treatment modality for medical or dental practitioners; however, it should be used carefully, under the supervision of an experienced medical professional [10,23,24]. The benefit of laser radiation—instead of using analgesic medications—relates to the fact that there are no systemic adverse effects for the patient.

Earlier studies have evaluated the effects of LLLT on reducing the pain caused by orthodontic ESs; however, the reported results are still contradictory. Some investigations supported the notion of analgetic effects [25,26,27,28,29,30,31,32,33,34], while other studies could not verify it [17,35,36,37]. Therefore, due to the controversial position of LLLT as a treatment modality in orthodontic pain, recent reviews on this topic indicated that further, well-designed randomized controlled trials are needed to clarify the role and usefulness of LLLT in the reduction of orthodontic pain [10,38,39,40,41,42,43]. Therefore, the aim of this clinical study was to assess the effect of LLLT in reducing the pain following orthodontic ES placement in individuals treated with fixed orthodontic appliances, using a randomized, double-blind placebo-controlled (RDBPC) split-mouth study design. Based on the literature, our initial hypothesis was that LLLT treatment is effective in reducing pain following orthodontic treatment, both corresponding to spontaneous pain and pain on mastication.

## 2. Materials and Methods

### 2.1. Study Setting, Inclusion and Exclusion Criteria, Sample Size Determination

Participants were included in the study from the pool of patients at the Department of Orthodontics and Pediatric Dentistry (Faculty of Dentistry, University of Szeged) requiring orthodontic treatment. The following inclusion criteria were set: (i) individuals aged between 18 and 50 years; (ii) completely erupted second molars without open interproximal contacts of the first molars; (iii) good general health, without the existence of systemic diseases; (iv) adequate oral hygiene and healthy periodontium; and (v) no previous orthodontic treatment. The following exclusion criteria were set: (i) those who did not fulfill the general inclusion criteria; (ii) patients who had undergone prior oral LLLT; (iii) those who were using painkillers or other NSAIDs; (iv) those who consumed tobacco products; and (v) pregnant or lactating women.

With the expectation of a mean ± SD of 25.0 ± 7.0 on the visual analogue scale (VAS) for pain readings and a mean reduction in pain by 30% due to LLLT—based on the previously published literature—a minimum required sample size of *n* = 32 (i.e., 16–16 for the LLLT-treated group and placebo group, respectively) was determined, with a split-mouth study enrollment ratio of 1:1, an alpha (α) set at 0.05, a beta (β) set at 0.2 and statistical power set at 0.8 (80%). Sample size calculations were carried out using the ClinCalc platform [44].

The study was carried out between 2021. 07. 05 and 2021. 12. 23, when the study was concluded after the sufficient number of participants were included. Our study adheres to the guidelines set by the Consolidated Standards of Reporting Trial (CONSORT) statement [45].

### 2.2. Ethical Considerations

This study was conducted in accordance with the Declaration of Helsinki (1975, last revised in 2013) and national and institutional ethical standards. Ethical approval for the study was obtained from the National Institute of Pharmacy and Nutrition, Department of Health Technology, Hungary (reference number: OGYÉI/38078-6/2020; approval date: 3 July 2020). Additionally, the Human Institutional and Regional Biomedical Research Ethics Committee, University of Szeged, was informed about the above-mentioned ethical approval and registered the study locally (reference number: 190/2020-SZTE; approval date: 14 December 2020).

Written informed consent was obtained from the patients who agreed to participate before the initiation of the study. They were briefed about the research objectives, privacy and confidentiality of their data, and they were made aware that their participation in the research was voluntary, and that they might withdraw from the study at any time.

### 2.3. Study Design

A randomly assigned, split-mouth design was used to avoid inter-individual biological variation between patients [46]. First, it was randomly determined whether the upper or lower jaw would be involved in the study, using cards labeled with “UPPER JAW” and “LOWER JAW”. After choosing the card (jaw) blindly, the actual dental arch in the mouth was further divided vertically, into right and left halves (quadrants) in each patient. One half was the experimental side (i.e., the test quadrant) and received laser therapy, while the other half was the placebo side (i.e., placebo quadrant), which received no laser therapy, only placebo therapy. The choice of the quadrants to receive laser treatment or placebo treatment was determined randomly using further cards with the labels “LEFT SIDE LASER” and “RIGHT SIDE LASER”. Patients were asked to choose from among the two cards. To achieve the double-blinded study design, patients and operator were blinded to the experimental and placebo sides. Patients were asked not to use anti-inflammatory or analgesic drugs during the study period.

### 2.4. Application Protocol

The placement of ESs (Dentalastic^®®^ separators, blue, Dentaurum^®®^, Ispringe, Germany) was performed for all patients on the first permanent molars (mesially and distally) in both quadrants of the randomly chosen lower or upper jaws. Then, the treatments were performed according to the randomly selected treatment types, i.e., one quadrant received the placebo, while the other one was treated with the laser in the same patient.

### 2.5. Laser Irradiation

**Figure 1:**
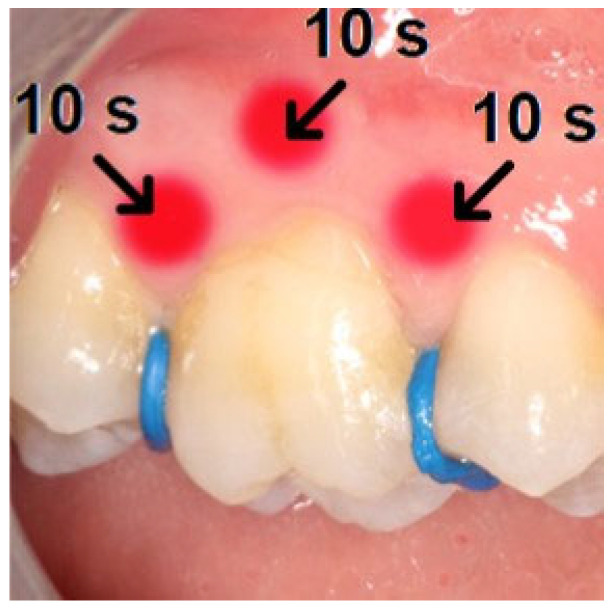
Locations of the LLLT and placebo treatments in the test and placebo sides.

A low-level medical gallium–aluminum–arsenide (GaAlAs) diode laser device (Medency Primo dental laser, Vicenza, Italy; wavelength 980 nm, power: 100 mW, producing 6 J of energy), with a continuous wave was used on the 1st permanent molars in the test quadrants. Using the split-mouth design, patients received only a single dose immediately after the separators’ placement. Specified points in each quadrant were treated from mesial, central and distal directions on the mucosa both buccally and lingually/palatinally (Figure 1), with 10–10 s for each, altogether for 60 s, producing 36 J of energy per molar. During the exposure, the laser tip was placed perpendicular to the long axis of the teeth, with close contact between the tip and mucosa.

During the placebo treatment session, a similar procedure to that of the laser treatment session was carried out but with no laser irradiation. Therefore, to control the patient’s pain-related behavior, and to maintain the blinded protocol, the handpiece was also held on the placebo treatment side without laser irradiation following a similar application protocol. In accordance with bio-safety standards, and to maintain the blinded protocol at the time of the laser irradiation and placebo treatments, both the patient and operator used goggles (safety glasses) designed to block the wavelength of the laser (980 nm), and earplugs were also used to block the typical sounds of laser activity. This way, neither the patient nor the operator could identify the type of the therapy for the respective quadrants; only the person who handled the equipment was aware of the allocation, i.e., the treatment groups. To avoid intra-operator variations, a single operator placed the ESs and applied the laser and placebo treatment for all patients.

### 2.6. Determination and Registration of Pain Levels

Patients received a code number on a separate sheet that contained the sex of the participant, date of birth (age), date of the treatment applications (elastics, treatments) and treatment location (jaw and side of laser treatment [test quadrant]), as well as the serial number of the patient, which ensured objective evaluation and analysis. Pain was registered and evaluated on a 2-page, standardized questionnaire filled out by each patient (in accordance with the two different types of treatments in the two quadrants of the upper or lower jaws). Patients were instructed to denote their pain sensation levels (which were the main outcome measure of the study) themselves, according to the localization (upper/lower jaw; right/left side) and the given appointments—at 0 h (h) (i.e., within 5 min of placing the ESs), at 6 h (h) (±30 min), at 12 h (±30 min), at 24 h (±30 min), at 48 h (±30 min) and at 72 h (±30 min) after treatment (laser/placebo)—in the resting position of the mandible (spontaneous pain) and during mastication, on the VAS scale, from 0 (no pain) to 100 (severe pain), found in the standardized questionnaire [47,48,49]. Besides this, the questionnaire contained information about the (serial) number of the patient and location (jaw) and date of the treatments. The pain level (the distance from zero to the mark) registered by the patient was measured using a universal digital caliper (MIB Messzeuge GMBH, Spangenberg, Germany). Following the measurements, a data table was prepared for statistical analyses.

### 2.7. Statistical Analysis

All continuous variables were expressed as means and standard deviations (mean ± SDs), whereas categorical variables were expressed as frequencies (*n*) and percentages (%). The normality of variables was tested using the graphical method (Q-Q diagram) and Shapiro–Wilk tests. Pain levels recorded in the LLLT-treated and placebo groups were compared using Welch’s independent-sample *t*-test. Statistical analyses were performed using SPSS Statistics version 22.0 (IBM Inc., Chicago, IL, USA). During the analyses, *p* values < 0.05 were considered statistically significant.

### 2.8. Trial Registration

The study has been registered on clinicaltrials.gov, under the identifier ID NCT06816537 (https://clinicaltrials.gov/study/NCT06816537) “The effect of low-level laser therapy to reduce pain caused by orthodontic separators: A randomized, double-blind placebo-controlled, split-mouth study (SZTE-DENT-2020-38078-6)” (date of registration: 7 February 2025, retrospectively registered).

## 3. Results

**Figure 2:**
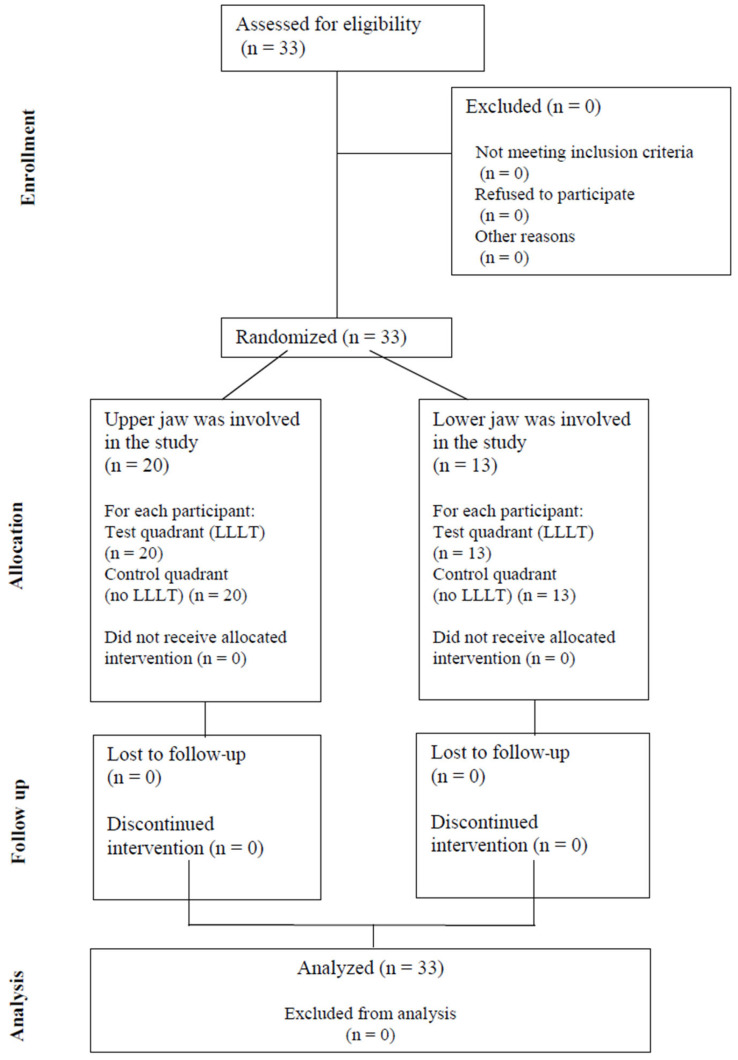
CONSORT flowchart representing the participants (*n* = 33) eligible and the final net number of participants (*n* = 33) in the study.

Overall, the net sample size of the study was *n* = 33 (12 male and 21 female; aged between 19 and 36 years [mean ± SD: 24.86 ± 6.63 years]); none of the involved volunteers had to be excluded, and all provided the necessary data required to participate in the study. Participating in this research did not result in the interruption of the patient’s treatment, and none (*n* = 0) of the participants required analgetic medication during the study period. None of the patients (*n* = 0) reported adverse effects or harms corresponding to the treatments. The CONSORT flowchart representing the participants of the study is shown in Figure 2., while the CONSORT checklist is presented in Supplementary Material S1.

There were no statistical differences in the general information of the individuals whose upper or lower jaw was involved in the study (*p* > 0.05). Regarding pain levels (as per recorded VAS values) in the resting position of the mandible, no significant differences were found between the test (LLLT-treated) and control (placebo) quadrants, at any time points after the interventions (*p* > 0.05 in all cases) (Table 1). During mastication, registered pain levels were significantly lower in the LLLT-treated (test) quadrants than in the placebo (control) quadrants (6 h: 9.29 ± 12.65 vs. 19.07 ± 20.99, *p* = 0.025; 24 h: 21.96 ± 21.11 vs. 37.19 ± 26.35, *p* = 0.012; 48 h: 28.01 ± 21.40 vs. 42.60 ± 26.29, *p* = 0.016), with the exception of the first and last measurements (Table 2).

**Figure 3:**
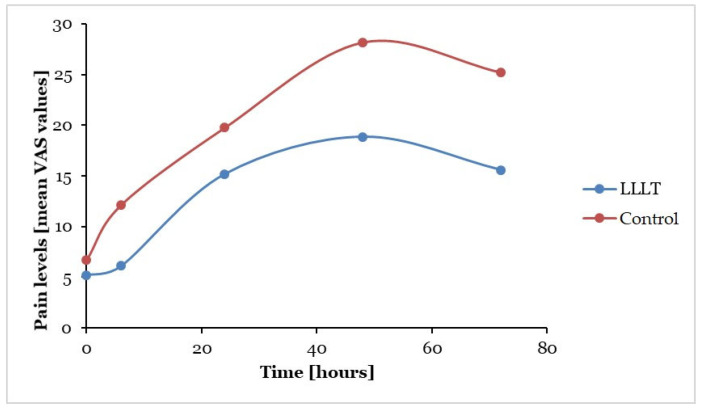
Changes in mean pain levels according to the different measurement times in the test (LLLT-treated) and control (placebo-treated) quadrants in the resting position of the mandible (spontaneous pain); LLLT: low-level laser therapy; VAS: visual analogue scale.

**Figure 4:**
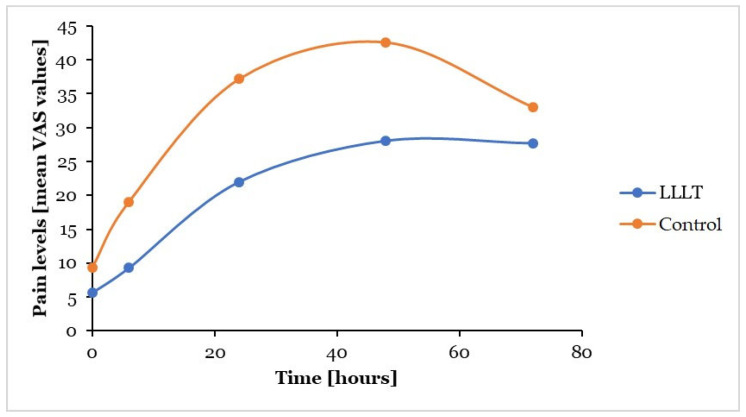
Changes in mean pain levels according to the different measurement times in the test (LLLT) and control (placebo-treated) quadrants during mastication; LLLT: low-level laser therapy; VAS: visual analogue scale.

When comparing the test and control quadrants, the reduction in pain levels caused by the applied LLLT was highest after 6 h, following applications both in the resting position of mandible (49%) and during mastication (51%) (Table 3). In the resting position of the mandible, the mean VAS values were lower in the test quadrants compared to the control sides at every measurement time (Figure 3). During mastication—similarly to the situation found in the resting position of the mandible—VAS values were lower in the test quadrants compared to control quadrants at every registration time (Figure 4).

## 4. Discussion

Orthodontic pain is based on the stretching and compressing forces acting on the periodontal ligaments and the surrounding bone structures by the orthodontic interventions. These forces result in changes in periodontal blood flow and consequently cause an inflammatory response through the releasing of various tissue materials and inflammatory mediators (e.g., prostaglandines, histamine). These inflammatory processes contribute to the hypersensitivity and enhanced pain sensation of free nerve endings during orthodontic treatments [41]. Orthodontic pain may cause considerable discomfort for patients, and the high pain levels experienced may lead to the interruption of orthodontic treatment. To avoid this unpleasant outcome, some sort of analgetic treatment modality should be offered for all patients. LLLT may be effective in reducing the pain during orthodontic treatment and after ES placement as well, which is the first intervention at the beginning of orthodontic treatment with a fixed appliance [20,25,26,27,30,39]. The analgetic effect of LLLT may be explained by its anti-inflammatory and neuronal effects, including stimulation of the cellular activity of neurons and lymphocytes, stabilization of membrane potentials and neurotransmitter release in the inflamed tissues [19]. A reduction in pain should potentially increase patient cooperation and, consequently, improve the outcomes of orthodontic treatment [5]. Furthermore, effective pain management would minimize the need for the use of anti-inflammatory drugs, consequently avoiding their systemic adverse effects, in addition to their negative effects on orthodontic treatment outcomes [9].

Laser therapy represents a non-surgical, minimally invasive, non-pharmacological method for medical interventions [41]. Most of the studies have concentrated on GaAlAs diode lasers with wavelengths ranging from 600 to 1000 nm, to investigate their effectiveness on orthodontic pain. However, because of the inconsistent laser parameters reported and large, inter-subject variations contributing to conflicting outcomes, there is still no consensus on the analgetic effects of LLLT [50,51,52]. The effects of inter-subject variation may be minimized when the individual is self-matched or self-controlled [53,54]. There are various influencing factors regarding the degree of pain, which may be classified as objective (e.g., age, sex, the applied forces) or subjective (e.g., the actual mental and psychical condition of the patient, stress, ethnicity, individual tolerance of pain, previous painful experiences) [5,55]. During the application of lasers in such studies, the confounding effects of these factors should be avoided. The use of the “split-mouth” technique—as applied in our study—may be a suitable method to obtain exact results considering individual pain control [53,54]. The level of pain in the test and control (placebo) quadrants can be compared in the same participant of the study group; thus, influencing factors—affecting the level of pain experienced by the individuals—may be avoided. Overall, to apply the split-mouth design in studies investigating orthodontic pain is justified and advantageous compared to parallel-design studies.

At the same time, in spite of the potential pain-reducing effect of LLLT, some limitations of this treatment should be considered, which were summarized in a systematic review by Inchingolo et al. (2023) [10]: LLLT may have a variable effectiveness (depending on the individual, method of application, choice of light parameters); results are postponed; it has depth limitations; there is a risk of misuse (when the operator is not experienced, there is a possibility of tissue damage); and there is the cost and affordability of the instrument and the treatment. Therefore, further studies are needed related to the effectiveness and safe use of this method [10]. Based on the currently available literature, the other published reviews also suggest that further research, with a better study design and appropriate sample size/statistical power, is needed to provide more reliable evidence for the clinical application of diode LLLT in relation to pain induced by orthodontic interventions [10,38,39,40,41,42,43,55].

When the RDBPC design is compared with other research designs, the level of evidence given by RDBPCs is the most reliable, and hence it is considered as the “gold standard” for comparison [56]. Randomization with blinding avoids reporting bias. Furthermore, when the expectations of the patient and/or the investigator may conceivably affect the outcome, then double blinding is especially important [46]. In addition, a placebo must be used to determine if any improvement in the treated group is due to the LLLT’s effect, rather than the act of being treated alone. Considering the information mentioned above, evaluating the activity of LLLT (GaAlAs laser, wavelength 980 nm) in reducing pain, using the RDBPC design, was the principal aim of the present study, including the assessment of the effect of a single dose of LLLT on spontaneous pain and pain during mastication, as well as pain caused by the placement of orthodontic separators.

The use of the VAS scale for the evaluation of pain intensity has been generally accepted in orthodontic studies, as it is a quick and simple method with good reliability and sensitivity, which is based on the self-reported perception of individual pain. The most important limitations of this method are that the questionnaire can be completed only in written (on paper or electronically) form, and it is not suitable for patients with physical disabilities or cognitive dysfunctions [48]. Furthermore, the inter-individual differences in the pain experience and tolerance levels may also affect the results considerably; this was also demonstrated in our study, as seen in the relatively high standard deviations, especially at the measurement time points “0 h” and “6 h”. Considering the results of this study, the analgetic effect of laser treatment existed when orthodontic elastomers were used at the beginning of orthodontic treatment to create enough space for the bands included in a fixed appliance. Most of the previous studies on the effectiveness of laser irradiation evaluated spontaneous pain only, while we assessed the analgetic effect of laser treatment on spontaneous pain (in the resting position of the mandible) and pain during mastication.

Several studies have evaluated the analgetic effect of LLLT during the first part of orthodontic interventions using interdental ESs. However, there were considerable differences in the age of participants, the equipment used, energy, frequency of treatment, lighting points, time points of measurement, duration of the study and design of the study, among others. For this reason, it is difficult to objectively compare all the published results appropriately, even in cases of similar examinations. Using different methodologies, the final results and conclusions were similar to ours in the studies published by Artés-Ribas et al. (2013), Nóbrega et al. (2013), Eslamian et al. (2014), Farias et al. (2016), Qamruddin et al. (2016), Marini et al. (2015), Muctar et al. (2021), Stein et al. (2015), Gupta et al. (2018) and Almallah et al. (2016) [25,26,27,28,29,30,31,32,33,34]. These studies found significant differences between the pain values of LLLT-treated and placebo-treated groups, showing the effectiveness of LLLT in reducing the orthodontic pain caused by ES placement. Several of the these studies—similarly to the present study—used a split-mouth technique with a placebo control [25,27,28,29,31,33,34]. Other studies, such as Lim et al. (1995), Furquim et al. (2015), AlSayed Hasan et al. (2018) and Oshagh et al. (2014), experienced some beneficial effect in pain reduction after LLLT treatment, but they did support that LLLT has relevant analgetic effects in the case of using ESs [17,35,36,37].

Among the mentioned studies, similarly to our conception, Nóbrega et al., Qamruddin et al., and Gupta et al. evaluated the spontaneous pain and pain during mastication as well, after using orthodontic ESs [26,29,33]. In the study of Nóbrega et al., the participants were divided into test and placebo groups by randomization, while Qamruddin et al. and Gupta et al. determined the laser-treated test and placebo-treated control quadrants using the split-mouth technique. Nóbrega et al. used laser treatment with a 830 nm wavelength laser, while Qamruddin et al. and Gupta et al. applied 940 nm wavelength in the test quadrants; to compare, our laser device had a wavelength of 980 nm. According to the results of Nóbrega et al., there were significantly more “0” VAS values in the LLLT-treated group than in the control group of patients, and the mean pain values were significantly lower in the test group than in the controls at every registration appointment, with the exception of the last (5th) day (when differences were not significant), in both spontaneous and occlusion situations. In the laser-treated group—apart from the 2 h appointment—the mean pain levels were significantly higher in occlusion than in rest [26]. Similarly, in our research, a higher level of pain was associated with chewing than in rest, in both test and placebo quadrants at every time point, a finding noted by Gupta et al. as well [33].

Gupta et al.—similarly to Qamruddin et al.—also experienced significant differences in pain values between the LLLT-treated and placebo-treated quadrants, favoring the group that received LLLT both in the resting position of the mandible (spontaneous pain) and during mastication [29,33]. Although we did not report statistically significant differences between the two sides in the resting position of the mandible, but values have shown a tendency to be lower in the test quadrants at every measurement time. Furthermore, except for the first and last measurement points, we also found significant differences between the quadrants in pain levels upon chewing. In our study, mean pain levels during mastication were significantly lower in the laser-treated test quadrants compared to the controls after 6 h following the intervention, and the same results were shown after 24 and 48 h as well. However, there was no statistically significant difference between the test and control sides after 5 min, but already after 72 h, however, the pain levels were lower in the test quadrants than in the control sides at both appointments.

Based on these findings, it can be supposed that the anti-inflammatory and neuronal changes caused by laser therapy need a specific time interval to show a statistically significant effect (i.e., 5 min was too short; however, 6 h seemed to be enough). However, in accordance with our results, the duration of this effect is limited, i.e., lasting for 2 days, as after 72 h, the patients experienced it less after the single application. Qamruddin et al. found a statistically significant reduction in spontaneous pain and pain on chewing in the LLLT-treated quadrants compared to controls for all seven days of their study, similarly to the results experienced by Gupta et al., except for the first appointment (T1, where the difference was not significant) during their 2-day study period [29,33].

In their single-blind, split-mouth clinical study, Muctar et al. found that the majority (85.7%) of the patients experienced pain on chewing after separation. Significant difference was found between the pain intensities at different time intervals for the laser group, and there was a significant difference regarding the pain intensity between the laser and control sites at baseline. The intensity of pain experienced in the laser-treated quadrants increased in most of the cases just after 72 h, while in the control quadrants, this occurred much sooner, after 24 h. Thus, LLLT was shown to be effective in decreasing pain and discomfort, although the study was performed only on six patients [31]. Martins et al. (2018) published that in the case of spontaneous pain, laser therapy had analgetic effects immediately when applying interdental ESs; although their study was placebo-controlled, it did not have the split-mouth study design; therefore, individual influencing factors should be considered [56].

According to the literature, orthodontic pain peaks seems to be at 24 h; this was supported by most of the studies performed with elastomeric separators [26,28,29,31,34,56]. Stein et al. also experienced the pain peak at the end of the first day of the study (although they used a younger patient group with early mixed dentition in their examinations), and they noticed that based on the previous studies the highest pain intensities could be experienced within 4–24 h of ES placement in adults [32]. Artés-Ribas et al. found the peak at 6–24 h after separator placement. Concurrently, Eslamian et al. found the highest pain values 30 h after ES placement, while Gupta et al. published 36 h, and Marini et al. experienced the peak 24–36 h after separation [25,27,30,33]. In our study, mean VAS values were the highest at the “48 h” appointments during mastication and the pain peaks at the same appointments—although with lower values—associated with spontaneous pain.

Orthodontic pain is associated with an inflammation in the periodontal tissues when inflammatory mediators (e.g., prostaglandines) are released, and these mediators reach their highest concentration during 24–48 h after the intervention [31]. Within this framework, the experiences of the participants in our study may be explained. Orthodontic studies evaluating the effect of LLLT show great differences regarding the location and number of places or frequency of the laser treatments. We treated the teeth between the separators in three places both on the buccal and oral sides. The method was the same in the study of Artés-Ribas et al. [25]. In other investigations, other setups were utilized, e.g., Qamruddin et al. treated only the buccal mucosa of the examined teeth in three places, similarly to Muctar et al., who also treated the buccal mucosa only [29,31]. Martins et al. applied the laser irradiation at 4–4 locations on the buccal and lingual sides [56]. In the study of Almallah et al., two treatment groups were set up; in one group, the teeth were treated once after the placement of the ESs, while the second group was treated twice: after placement of the ESs and subsequently, again after 24 h. In this study, there were 4–4 treatment places by quadrants: near the mesial and distal surfaces of the first molars, the mesial surfaces of the second premolars and the distal surfaces of the second molars [34].

In all of the studies—similarly to our method—the VAS scale filled out by the participants was used for the evaluation of pain levels. However the registration times for the experience of pain and the studies overall were considerably different. Registration appointments were determined similarly to those of ours, e.g., by Artés-Ribas et al. and AlSayed Hasan et al. [25,36], while others groups have chosen longer periods or other ranges for their studies [26,27,28,29,30,31,32,33,34,56].

The evidence for the beneficial effect of LLLT in the adult population is supported by the results of our findings, i.e., that the reduction of pain was better in the treated quadrants compared to the controls; considering the measurement times of 6 h, 24 h and 48 h, the level of pain in the treated quadrants was reduced by 23–49% in the resting position of the mandible and by 34–51% during mastication. Although the best analgetic effect was experienced after 6 h following the LLLT, both in the resting position of the mandible and during mastication, the treatment still seems to be effective at the 48th hour after treatment, where the difference was significant between the two quadrants in the level of pain during mastication.

The results of our findings should be interpreted in the context of this study’s limitations, such as the single-center nature our study, performed at a clinical center offering tertiary-care health facilities, which may introduce selection bias and limit the generalizability of our findings. In addition to the limitations associated with the use of the VAS pain measurement method, the differences between their experiences, either during spontaneous pain or pain associated with mastication, might be explained by certain influencing factors existing in the participants of this study, which cannot be disclosed even with the use of the split-mouth method, such as typse of orthodontic anomalies (e.g., bite, crowding existing, other anomalies that may be present in one involved quadrant but not in the other). Furthermore, our study population included adult subjects only: in childhood—even when comparing children of identical ages—the condition of tissues and their reactivity to LLLT treatment may differ considerably, which could render the measured results uncertain. Furthermore, the filling out of the pain questionnaire form (i.e., administration) by children is less reliable, even if it is carried out with the aid of a parent/guardian (in fact, it may be a considerable influencing factor, introducing bias). The current study employed a single type of laser, utilizing a single dose/energy; to better establish the nature of the LLLT treatment’s effects, and the dose-response relationship, the use of multiple different laser devices and varied energy settings during treatments should be carried out in further studies.

In light of the recent reviews published in the literature regarding the necessity for further studies, and the heterogeneity presented in the above articles discussed, it is essential to establish more uniform, consequential and reproducible protocols to allow for an optimal comparisons between studies, thus to accurately determine the role and optimal use of laser therapy in reducing orthodontic pain in both adult and pediatric populations.

## 5. Conclusions

Increased pain levels due to orthodontic treatment with fixed appliances (including the placement of elastic interdental separators) may result in the suspending of the treatment. Based on this study, continuously applied LLLT with 6 J of energy may have beneficial effects as an analgesic treatment modality for pain caused by orthodontic elastomeric separators, used at the beginning of treatment with fixed appliances in adult patients. The pain-alleviating effects of LLLT were especially prominent during mastication and after 6–48 h following the separation procedure. In our corresponding adult population, pain reduction was the most expressed after 6 h following placement of separators, both in spontaneous pain and pain on mastication. LLLT may be a promising way in everyday clinical practice to reduce painful sensations and to increase treatment success and comfort.

## Figures and Tables

**Table 1:** Pain values measured on the visual analogue scale (VAS) in the LLLT-treated and placebo quadrants at the given time points for the resting position of the mandible (spontaneous pain).

	VAS Values (Mean ± SD)				
Measurement Time	0 h	6 h	24 h	48 h	72 h
Test quadrants (LLLT-treated)	5.23 ± 11.27	6.13 ± 10.23	15.18 ± 18.45	18.85 ± 21.09	15.58 ± 18.58
Placebo quadrants	6.68 ± 12.71	12.13 ± 17.19	19.76 ± 24.08	28.19 ± 27.95	25.22 ± 27.88
*p*-value	0.624	0.092	0.389	0.103	0.103

LLLT: low-level laser therapy; VAS: visual analogue scale; SD: standard deviation.

**Table 2:** Pain values measured on the visual analogue scale (VAS) in the LLLT-treated and placebo quadrants at the given time points, during mastication.

	VAS Values (Mean ± SD)				
Measurement Time	0 h	6 h	24 h	48 h	72 h
Test quadrants (LLLT-treated)	5.63 ± 11.89	9.29 ± 12.65	21.96 ± 21.11	28.01 ± 21.40	27.65 ± 23.19
Placebo quadrants	9.34 ± 14.62	19.07 ± 20.99	37.19 ± 26.35	42.60 ± 26.29	33.08 ± 26.44
*p*-value	0.265	**0.025**	**0.012**	**0.016**	0.378

LLLT: low-level laser therapy; VAS: visual analogue scale; SD: standard deviation; *p*-values below 0.05 are shown in **boldface**.

**Table 3:** Reduction in pain level in the test quadrants compared to the placebo quadrants in the resting position of the mandible and during mastication (Δ%).

	0 h	6 h	24 h	48 h	72 h
Resting position (spontaneous pain)	21.79%	49.47%	23.15%	34.82%	38.12%
During mastication	39.49%	51.29%	40.95%	34.24%	16.42%

## Data Availability

All data generated during the study are presented in this paper.

## Supplementary Materials

The following supporting information can be downloaded at: https://www.mdpi.com/article/10.3390/dj13050181/s1, Supplementary Material S1 (CONSORT checklist).

## References

[1] Krishnan V. (2007). Orthodontic pain: From causes to management—A review. Eur. J. Orthod..

[2] Polat O., Karaman A.I. (2005). Pain control during fixed orthodontic appliance therapy. Angle Orthod..

[3] Bergius M., Kiliaridis S., Berggren U. (2000). Pain in orthodontics. J. Orofac. Orthop..

[4] Polat O. (2007). Pain and discomfort after orthodontic appointments. Semin. Orthod..

[5] Brown D.F., Moerenhout R.G. (1991). The pain experience and psychological adjustment to orthodontic treatment of preadolescents, adolescents, and adults. Am. J. Orthod. Dentofacial. Orthop..

[6] Wang J., Jian F., Chen J., Ye N.S., Huang Y.H., Wang S., Huang R.H., Pei J., Liu P., Zhang L. (2012). Cognitive behavioral therapy for orthodontic pain control: A randomized trial. J. Dent. Res..

[7] Farzanegan F., Zebarjad S.M., Alizadeh S., Ahrari F. (2012). Pain reduction after initial archwire placement in orthodontic patients: A randomized clinical trial. Am. J. Orthod. Dentofacial Orthop..

[8] Angelopoulou M.V., Vlachou V., Halazonetis D.J. (2012). Pharmacological management of pain during orthodontic treatment: A meta-analysis. Orthod. Craniofac. Res..

[9] Knop L.A., Shintcovsk R.L., Retamoso L.B., Ribeiro J.S., Tanaka O.M. (2012). Non-steroidal and steroidal anti-inflammatory use in the context of orthodontic movement. Eur. J. Orthod..

[10] Inchingolo F., Inchingolo A.M., Latini G., Del Vecchio G., Trilli I., Ferrante L., Dipalma G., Palermo A., Inchingolo A.D. (2023). Low-Level Light Therapy in Orthodontic Treatment: A Systematic Review. Appl Sci..

[11] Nalcaci R., Cokakoglu S. (2013). Lasers in orthodontics. Eur. J. Dent..

[12] Abdildin Y., Tapinova K., Jyeniskhan N., Viderman D. (2023). High-intensity laser therapy in low back pain management: A systematic review with meta-analysis. Lasers Med. Sci..

[13] Sant’Anna E.F., de Souza Araújo M.T., Nojima L.I., da Cunha A.C., da Silveria B.L., Marquezan M. (2017). High-intensity laser application in orthodontics. Dental Press J. Orthod..

[14] Harris D.M. (1991). Biomolecular mechanism of laser biostimulation. J. Clin. Laser Med. Surg..

[15] Harazaki M., Isshiki Y. (1997). Soft laser irradiation effects on pain reduction in orthodontic treatment. Bull. Tokyo Dent. Coll..

[16] Fukui T., Harazaki M., Muraki K., Sakamoto T., Isshiki Y., Yamaguchi H. (2002). The evaluation of laser irradiated pain reductive effect by occlusal force measurement. Orthod. Waves.

[17] Lim H.M., Lew K.K., Tay D.K. (1995). A clinical investigation of the efficacy of low level laser therapy in reducing orthodontic postadjustment pain. Am. J. Orthod. Dentofacial Orthop..

[18] Saito S., Mikikawa Y., Usui M., Mikawa M., Yamasaki K., Inoue T. (2002). Clinical application of a pressure sensitive occlusal sheet for tooth pain time dependent pain associated with a multi bracket system and the inhibition of pain by laser irradiation. Orthod. Waves.

[19] Turhani D., Scheriau M., Kapral D., Benesch T., Jonke E., Bantleon H.P. (2006). Pain relief by single low level laser irradiation in orthodontic patients undergoing fixed appliance therapy. Am. J. Orthod. Dentofacial Orthop..

[20] Tortamano A., Lenzi D.C., Haddad A.C., Bottino M.C., Dominguez G.C., Vigorito J.W. (2009). Low level laser therapy for pain caused by placement of the first orthodontic archwire: A randomized clinical trial. Am. J. Orthod. Dentofacial Orthop..

[21] Youssef M., Ashkar S., Hamade E., Gutknecht N., Lampert F., Mir M. (2008). The effect of low level laser therapy during orthodontic movement: A preliminary study. Lasers Med. Sci..

[22] Li X., Tang Y., Chen Y. (2010). Interventions for pain during fixed orthodontic appliance therapy. A systematic review. Angle Orthod..

[23] Dima R., Tieppo F.V., Towery C., Davani S. (2018). Review of Literature on Low-Level Laser Therapy Benefits for Nonpharmacological Pain Control in Chronic Pain and Osteoarthritis. Altern. Ther. Health Med..

[24] Grassi F.R., Ciccolella F., D’Apolito G., Papa F., Iuso A., Trentadue R., Nardi G.M., Scivetti M., De Matteo M., Silvestris F. (2011). Effect of Low-Level Laser Irradiation on Osteoblast Proloferation and Bone Formation. J. Biol. Regul. Homeost. Agents.

[25] Artés-Ribas M., Arnabat-Dominguez J., Puigdollers A. (2013). Analgesic effect of a low-level laser therapy (830 nm) in early orthodontic treatment. Lasers Med. Sci..

[26] Nóbrega C., da Silva E.M., de Macedo C.R. (2013). Low-level laser therapy for treatment of pain associated with orthodontic elastomeric separator placement: A placebo-controlled randomized double-blind clinical trial. Photomed. Laser Surg..

[27] Eslamian L., Borzabadi-Farahani A., Hassanzadeh-Azhiri A., Badiee M.R., Fekrazad R. (2014). The effect of 810-nm low-level laser therapy on pain caused by orthodontic elastomeric separators. Lasers Med. Sci..

[28] Farias R.D., Closs L.Q., Miguens S.A. (2016). Evaluation of the use of low-level laser therapy in pain control in orthodontic patients: A randomized split-mouth clinical trial. Angle Orthod..

[29] Qamruddin I., Alam M.K., Fida M., Khan A.G. (2016). Effect of a single dose of low-level laser therapy on spontaneous and chewing pain caused by elastomeric separators. Am. J. Orthod. Dentofacial Orthop..

[30] Marini I., Bartolucci M.L., Bortolotti F., Innocenti G., Gatto M.R., Bonetti G.A. (2015). The effect of diode superpulsed low-level laser therapy on experimental orthodontic pain caused by elastomeric separators: A randomized controlled clinical trial. Lasers Med. Sci..

[31] Muctar S.A., Marouf A.A., Awooda E.M. (2021). The effectiveness of low-level laser therapy on controlling pain and discomfort during separator placement before fixation of orthodontic appliances. Indian J. Pain.

[32] Stein S., Korbmacher-Steiner H., Popovic N., Braun A. (2015). Pain reduced by low-level laser therapy during use of orthodontic separators in early mixed dentition. J. Orofac. Orthop..

[33] Gupta S., Ahuja S., Bhambri E., Sharma S., Sharma R., Kalia H. (2018). Evaluating the effect of low-level laser therapy on pain induced by orthodontic separation: A randomized split-mouth clinical trial. Lasers Dent. Sci..

[34] Almallah M.M., Almahdi W.H., Hajeer M.Y. (2016). Evaluation of Low Level Laser Therapy on Pain Perception Following Orthodontic Elastomeric Separation: A Randomized Controlled Trial. J. Clin. Diagn. Res..

[35] Furquim R.D., Pascotto R.C., Rino Neto J., Cardoso J.R., Ramos A.L. (2015). Low-level laser therapy effects on pain perception related to the use of orthodontic elastomeric separators. Dental Press J. Orthod..

[36] AlSayed Hasan M.M.A., Sultan K., Hamadah O. (2018). Evaluating low-level laser therapy effect on reducing orthodontic pain using two laser energy values: A split-mouth randomized placebo-controlled trial. Eur. J. Orthod..

[37] Oshagh M., Najafi H.Z., Bahramnia F., Gharesi-Fard B. (2014). Comparison of the analgesic effect of Ibuprofen and pulsed low-level laser in reducing pain after orthodontic separator placement and evaluation of the changes in the sulcular pain especially prostaglandin E2 level. J. Dent. Lasers.

[38] Grajales M., Rios-Osorio N., Jimenez-Peňa O., Mendez-Sanches J., Sanchez-Fajardo K., García-Berdomo H.A. (2023). Effectiveness of photobiomodulation with low-level lasers on the acceleration of orthodontic tooth movement: A systematic review and meta-analysis of split-mouth randomised clinical trials. Lasers Dent. Sci..

[39] He W.L., Li C.J., Liu Z.P., Sun J.F., Hu Z.A., Yin X., Zou S.J. (2013). Efficacy of low-level laser therapy in the management of orthodontic pain: A systematic review and meta-analysis. Lasers Med. Sci..

[40] Gkantidis N., Mistakidis I., Kouskoura T., Pandis N. (2014). Effectiveness of non-conventional methods for accelerated orthodontic tooth movement: A systematic review and meta-analysis. J. Dent..

[41] Shi Q., Yang S., Jia F., Xu J. (2015). Does low level laser therapy relieve the pain caused the placement of the orthodontic separators—A meta-analysis. Head Face Med..

[42] Chong R., McGrath C., Yang Y. (2015). The effectiveness of low-level diode laser therapy on orthodontic pain management: A systematic review and meta-analysis. Lasers Med. Sci..

[43] Li F.J., Zhang J.Y., Zeng X.T., Guo Y. (2015). Low-level laser therapy for orthodontic pain: A systematic review. Lasers Med. Sci..

[44] ClinCalc.com. https://clincalc.com/stats/SampleSize.aspx.

[45] Schulz K.F., Altman D.G., Moher D., The CONSORT Group (2010). CONSORT 2010 Statement: Updated guidelines for reporting parallel group randomised trials. BMJ.

[46] Antczak-Bouckoms A.A., Tulloch J.F., Berkey C.S. (1990). Splitmouth and cross-over designs in dental research. J. Clin. Periodontol..

[47] Hawker G.A., Mian S., Kendzerska T., French M. (2011). Measures of adult pain. Visual Analog Scale for Pain (VAS Pain), Numeric Rating Scale for Pain (NRS Pain), McGill Pain Questionnaire (MPQ) Short–Form McGill Pain Questionnaire (SF-MPQ), Chronic Pain Grade Scale (CPGS) Short Form-36 Bodily Pain Scale (SF-36 BPS) and Measure of Intermittent and Constant Osteoarthritis Pain (ICOAP). Arthritis Care Res..

[48] Vitale M.C., Falzinella C., Sfondrini M.F., Defabianis P., Scribante A. (2023). The Use of Questionnares in Pain Assassment during Orthodontic Treatments: A Narrative Review. Medicina.

[49] Kazi A.M., Khalid W. (2012). Questionnaire designing and validation. J. Pak. Med. Assoc..

[50] Sun G., Tunér J. (2004). Low-level laser therapy in dentistry. Dent. Clin. N. Am..

[51] Walsh L.J. (1997). The current status of low level laser therapy in dentistry, Part 1. Soft tissue applications. Aust. Dent. J..

[52] Kert J., Rose L. (1989). Clinical Laser Therapy—Low Level Laser Therapy.

[53] Lesaffre E., Philstrom B., Needleman I., Worthington H. (2009). The design and analysis of split mouth studies: What statisticians and clinicians should know. Stat. Med..

[54] Farzan A., Khaleghi K. (2021). The effectiveness of low-level laser therapy in pain induced by orthodontic separator placement: A systematic review. J. Lasers Med. Sci..

[55] Misra S. (2012). Randomized double blind placebo control studies, the “Gold Standard” in intervention based studies. Indian J. Sex. Transm. Dis..

[56] Martins I.P., Martins R.P., Caldas S.G.F.R., dos Santos-Pinto Buschang P.H., Pretel H. (2019). Low-level laser therapy (830nm) on orthodontic pain: Blinded randomized clinical trial. Lasers Med. Sci..

